# A novel genetic engineering platform for the effective management of biological contaminants for the production of microalgae

**DOI:** 10.1111/pbi.12564

**Published:** 2016-05-28

**Authors:** Maribel M. Loera‐Quezada, Marco Antonio Leyva‐González, Gilberto Velázquez‐Juárez, Lenin Sanchez‐Calderón, Mauro Do Nascimento, Damar López‐Arredondo, Luis Herrera‐Estrella

**Affiliations:** ^1^ Laboratorio Nacional de Genómica para la Biodiversidad Unidad de Genómica Avanzada del Centro de Investigación y de Estudios Avanzados del Instituto Politécnico Nacional Guanajuato México; ^2^ StelaGenomics México S de RL de CV Guanajuato México; ^3^ Instituto de Investigaciones en Biodiversidad y Biotecnología Buenos Aires Argentina; ^4^ Instituto de Ecología A.C. Xalapa, Veracruz Mexico

**Keywords:** phosphite metabolism, microalgae, contamination, open ponds

## Abstract

Microalgal cultivation that takes advantage of solar energy is one of the most cost‐effective systems for the biotechnological production of biofuels, and a range of high value products, including pharmaceuticals, fertilizers and feed. However, one of the main constraints for the cultivation of microalgae is the potential contamination with biological pollutants, such as bacteria, fungi, zooplankton or other undesirable microalgae. In closed bioreactors, the control of contamination requires the sterilization of the media, containers and all materials, which increases the cost of production, whereas open pond systems severely limits the number of species that can be cultivated under extreme environmental conditions to prevent contaminations. Here, we report the metabolic engineering of *Chlamydomonas reinhardtii* to use phosphite as its sole phosphorus source by expressing the *ptxD* gene from *Pseudomonas stutzeri* WM88, which encodes a phosphite oxidoreductase able to oxidize phosphite into phosphate using NAD as a cofactor. Engineered *C. reinhardtii* lines are capable of becoming the dominant species in a mixed culture when fertilized with phosphite as a sole phosphorus source. Our results represent a new platform for the production of microalgae, potentially useful for both closed photobioreactors and open pond systems without the need for using sterile conditions nor antibiotics or herbicides to prevent contamination with biological pollutants.

## Introduction

Microalgae have the potential to produce a broad range of products with numerous commercial applications (Borowitzka, 2013; Pignolet *et al*., 2013; Singh and Gu, 2010). Currently, open pond cultivation (mainly raceway ponds) is the most promising system for the production of biomass or metabolites from microalgae because their construction and operation is easier and less expensive than the different types of closed production systems (Mata *et al*., 2010; Schenk *et al*., 2008; Ugwu *et al*., 2008). In fact, over 90% of microalgal biomass production worldwide is currently achieved in large raceway ponds (Zhang *et al*., 2014), with *Spirulina*,* Chlorella* and *Dunaliella* as the three species that dominate the market, with an annual production of 3000, 2000 and 1200 tonnes of dry weight, respectively (Brennan and Owende, 2010). It has been argued that open raceway pond production is more environmentally sustainable, as their operation requires less energy than closed reactor systems (Smith *et al*., 2010). The commercial success of these three microalgae species is due to their ability to tolerate extreme environmental conditions, such as high alkalinity (*Spirulina*), high salinity (*Dunaliella*) and high nutrient concentrations (*Chlorella*) (Pulz and Gross, 2004; Rawat *et al*., 2013; Varfolomeev and Wasserman, 2011). Extreme environmental conditions significantly reduce one of the most inherent risks of commercial algae culture, namely the contamination by ‘weedy’ microalgae, bacteria and zooplankton, which can lead to losses in biomass and productivity (Bínová *et al*., 1998; Borowitzka, 2013; Day *et al*., 2012; Letcher *et al*., 2013; Smith and Crews, 2014; Wang *et al*., 2013). However, the strategy for using extreme abiotic environments to reduce the risks of contamination by undesirable competitors excludes the majority of microalgae species for the production of biomass or derived bioproducts. Therefore, to cultivate microalgal species unable to grow in extreme culture conditions in open ponds, a range of methods have been proposed and in some cases implemented to reduce or prevent the impact of contamination, such as early harvesting before serious loss of the biomass or the use of a number of chemical, biological and physical treatments to reduce the risk of contamination (Carney and Lane, 2014; Kazamia *et al*., 2014; Méndez *et al*., 2012; Moreno‐Garrido and Cañavate, 2000; Wang *et al*., 2013). For instance, the company Saphire Energy Inc. uses qPCR to monitor pest contamination to optimize the time of fungicide treatment (Shurin *et al*., 2013). However, this practice can become expensive and detection is often too late resulting in important yield losses. As an alternative, closed systems of different shapes and architectures have been designed to allow the production of a larger number of microalgae species under sterile or semi‐sterile conditions (Mata *et al*., 2010; Schenk *et al*., 2008; Ugwu *et al*., 2008). However, the need of sterile conditions, in both the reactor and the cultivation media, significantly increases the operation costs of closed bioreactors (Grobbelaar, 2012; McBride *et al*., 2014).

To date, much of the research aimed at commercializing algal bioproducts has focused primarily on using genetics and metabolic engineering to maximize the yield of value‐added products, such as oils and pigments (Cordero *et al*., 2011; Liu *et al*., 2013; Trentacoste *et al*., 2013; Yu *et al*., 2011; Zeng *et al*., 2011)). However, no attempts have been made for managing contamination through metabolic engineering to generate microalgae capable of outcompeting undesirable microalgae or other microorganisms for restricted resources. We postulate that an effective way to create a selective environment for favouring monocultures of selected algal lineages or consortiums of microalgae species with desirable properties, without compromising growth and productivity, is the use of metabolic engineering to design microalgae strains capable of converting a nonmetabolizable source of an essential nutrient into a chemical form that can be easily incorporated into their metabolism. These metabolically engineered organisms will have a competitive advantage over competitors when grown on a media supplemented with the nonmetabolizable form of the selected essential nutrient, allowing a better control of contaminations in open systems and reducing the need for sterile conditions in closed reactors.

The vast majority of organisms can only use phosphate (Pi) as the source of phosphorus (P) for metabolic processes, but not other reduced chemical forms of P such as phosphite (Phi) or hypophosphite (HPhi) (Morton *et al*., 2005; Pasek, 2008; Ruthbaum and Baille, 1964; Schroetter *et al*., 2006; White and Metcalf, 2007). However, some bacterial isolates have been shown to encode operons that allow them to utilize Phi and/or HPhi as sole sources of P (Casida, 1960; Hirota *et al*., 2012; Metcalf and Wolfe, 1998; Poehlein *et al*., 2013; Schink *et al*., 2002; Wilson and Metcalf, 2005; Yang and Metcalf, 2004). The best‐characterized phosphite oxidation pathway is that of *Pseudomonas stutzeri WM88,* which consists of a five‐gene operon designated *ptxABCDE* (White and Metcalf, 2007). The genes *ptxABC* encode an ABC‐type phosphite transporter, the gene *ptxD* encodes an NAD‐dependent phosphite oxidoreductase, and the gene *ptxE* encodes a putative regulatory protein.

In contrast to prokaryotes, no evidence exists suggesting that eukaryotic organisms have the required genes for the metabolism of any P reduced compound. In fact, it has been extensively documented that plants cannot metabolize Phi (Ávila *et al*., 2013; Berkowitz *et al*., 2013) and more recently, we showed that a number of microalgae species, including *Chlamydomonas reinhardtii*,* Ettlia oleoabundans* and *Botryococcus braunii,* are unable to use Phi as a sole P source (Loera‐Quezada *et al*., 2015). To develop microalga that can be used to design systems to prevent undesirable contaminations in open or closed cultivation systems, we designed a system to make cells able to metabolize Phi, a nonmetabolizable compound, as a sole P source. This strategy has already been successfully shown to be effective by the production of transgenic plants capable of using Phi as a sole source of P, which can outcompete weeds when fertilized with Phi instead of Pi (López‐Arredondo and Herrera‐Estrella, 2012).

In this study, we report the generation of transgenic *C. reinhardtii* lines capable of using Phi as the sole P source. These transgenic lines became the dominant species in mixed cultures, showing that the capacity of metabolizing Phi can provide a competitive advantage to the engineered strains, which is crucial to prevent or severely limit the invasion of open or closed culture systems by undesirable microalgae and/or many other microorganisms.

## Results

### Generation and characterization of transgenic *Chlamydomonas reinhardtii* lines

To test whether we could engineer the wild‐type *C. reinhardtii* CC‐125 (CrWT) strain to use Phi as a P source, we designed and introduced into the nuclear genome of this microalgae a gene construct containing a codon‐optimized version of the *ptxD* coding sequence under the control of the Hsp70A‐RbcS2 promoter (Experimental Procedures section). The plasmid used for transformation also contained a bleomycin resistance gene that was used as a selectable marker in the *C. reinhardtii* transformation experiments. We obtained 312 independent bleomycin‐resistant clones from which five of them were capable of growing in plates containing Phi as a sole P source.

**Figure 1:**
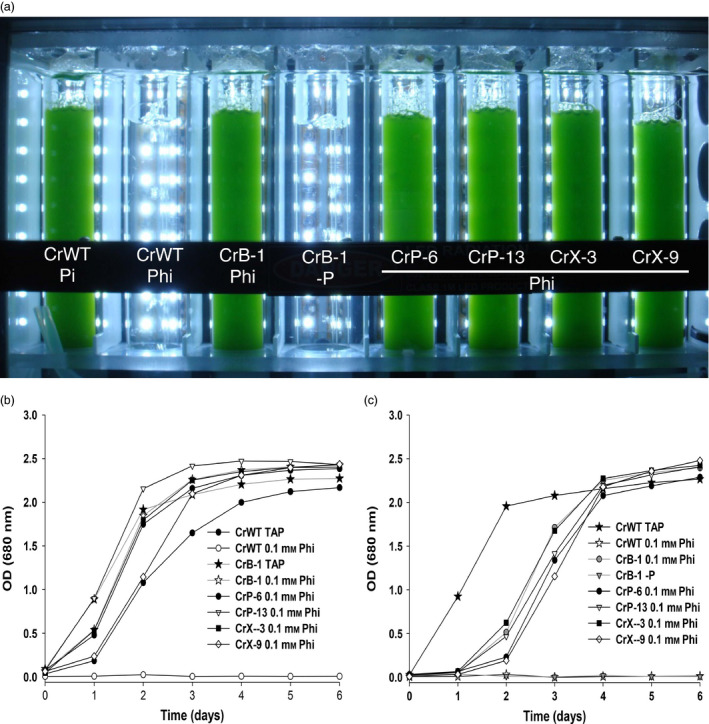
Growth of *Chlamydomonas reinhardtii* transgenic lines using phosphite as a phosphorus source. Growth of positive PTXD transgenic lines of *C. reinhardtii* (CrB‐1, CrP‐6, CrP‐13, CrX‐3, CrX‐9) in Tris‐Acetate (TA) media that either did not contain phosphorus (‐P) or was supplemented with 0.1 mm phosphate (Pi), or phosphite (Phi) as a P source. P‐starved (a,c) and P‐replete (b) cells were used as the inoculum for the experiments in which the optical density (OD) at 680 nm was measured every day for 6 days. Cultures were performed using a photobioreactor (Multi‐Cultivator MC 1000) at a light intensity of 250 μmol photons/m^2^/s, 28 °C and bubbled with air. The wild‐type *C. reinhardtii* CC‐125 (CrWT) strain was used as a control.

*Chlamydomonas reinhardtii* lines able to grow on plates with Phi as a sole P source, denominated CrB‐1, CrP‐6, CrP‐13, CrX‐3 and CrX‐9 lines, were then studied to determine the functional expression of the *ptxD* gene by directly culturing them in liquid medium containing Phi as the sole P source. For this purpose, we tested the growth of cells harvested from media containing 1 mm Pi (P‐replete cells) or from media devoid of a P source (P‐starved cells) in medium supplemented with 0.1 mm Phi as P source in a Multicultivator photobioreactor (MC 1000, Qubit Systems; Qubit Biology Inc. Kingston, Ontario, Canada) (Figure 1a). We tested P‐replete and P‐starved cells because we have previously observed that cells grown in 1 mm Pi are able to sustain significant growth in media lacking a P source (Loera‐Quezada *et al*., 2015). We observed that after 6 days of cultivation in media containing Phi as a sole P source the putative *ptxD* transgenic lines were all capable of growing with a growth rate similar to that of the WT or the same transgenic lines in media containing Pi as a P source (Figure 1; compare panel b versus c). Interestingly, we found that, although cultures inoculated with both P‐starved and P‐replete cells reached the stationary phase at a similar time, P‐starved cells presented a longer lag phase in comparison with that observed for the growth kinetics of P‐replete cells (Figure 1b,c)). This behaviour suggested that the P reserves, accumulated in the previous growth cycle of P‐replete cells, could favour a faster entry into the exponential growth phase in medium supplemented with Phi as a P source, compared with cells that needed to first convert enough Phi into Pi to initiate rapid cell growth.

**Figure 2:**
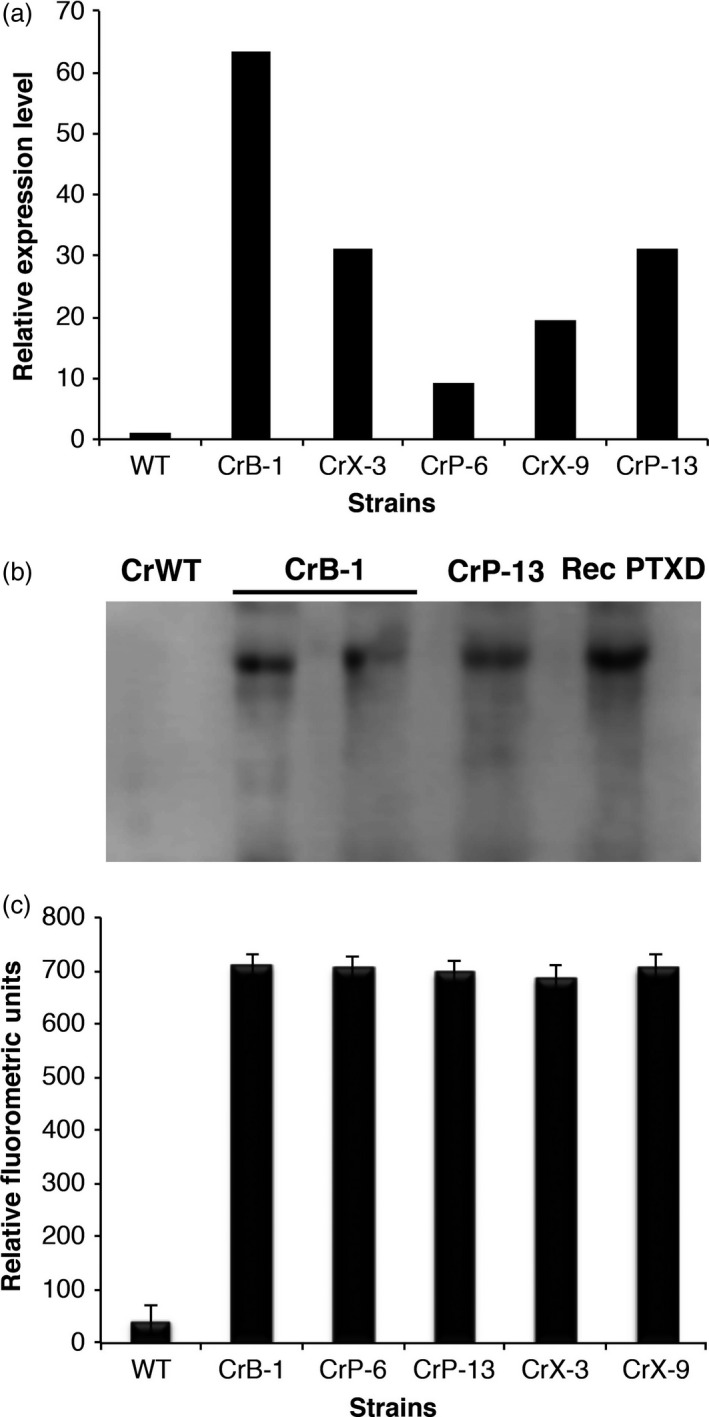
Characterization of *Chlamydomonas reinhardtii* transgenic lines. Positive *C. reinhardtii* transgenic lines (CrB‐1, CrP‐6, CrP‐13, CrX‐3, CrX‐9) were subjected to biochemical and molecular assays to determine the mRNA level and presence and functionality of the PTXD enzyme. For real‐time qPCR analysis (a), the relative expression levels of the *ptxD* transcripts were normalized in reference to that of the actin mRNA of the wild‐type *C. reinhardtii* CC‐125 (CrWT). In Western blot analysis (b), a recombinant version of the PTXD protein was purified from *E. coli* extracts and used as a positive control. The concentration of the protein was determined with a Bradford assay, and dilutions were generated to load the same amount of total protein. A monoclonal antibody specific for PTXD was used. The enzymatic activity (c), expressed in relative fluorometric units, was determined using a fluorometric assay. Statistical analysis was performed using a one way ANOVA (*P* < 0.05). The asterisk represents a significant difference.

To determine the expression of the *ptxD* gene and the presence of functional PTXD in the *C. reinhardtii* transgenic strains, we performed RT‐qPCR, Western blot assays and enzymatic activity assays to detect the *ptxD* transcript and the corresponding protein. As shown in Figure 2a, the *ptxD* transcript was only detected in the transgenic lines but not in the WT untransformed control. As expected from random insertions in the *C. reinhardtii* genome, we found differences in the *ptxD* transcript levels among the different transgenic lines, with line CrB‐1 having the highest amount of *ptxD* mRNA. We performed Western blot analysis of transgenic lines CrB‐1 and CrP‐13 using a monoclonal antibody specific for PTXD. As expected, no signal of the PTXD protein was observed in the WT control, whereas in both CrB‐1 and CrP‐13 transgenic lines, the signals corresponding to PTXD were present (Figure 2b). We determined PTXD activity in several *C. reinhardtii* transgenic strains, using an optimized fluorometric protocol previously reported (Berkowitz *et al*., 2011). We found that strains CrB‐1, CrP‐6, CrP‐13, CrX‐3 and CrX‐9 showed significant levels of PTXD activity, which correlated with their capability to metabolize Phi (Figure 2c). These assays corroborated that all the *C. reinhardtii* transgenic lines capable of using Phi as a P source do express the *ptxD* gene and contain an active PTXD protein. Although we detected significant differences in transcript levels between the different transgenic lines, these differences were not reflected in the protein or enzymatic activity levels detected by Western blot and enzymatic assays. Additionally, a visual inspection of cells from the transgenic lines confirmed that there were no significant differences in morphology, size and shape in reference to the CrWT strain.

**Figure 3:**
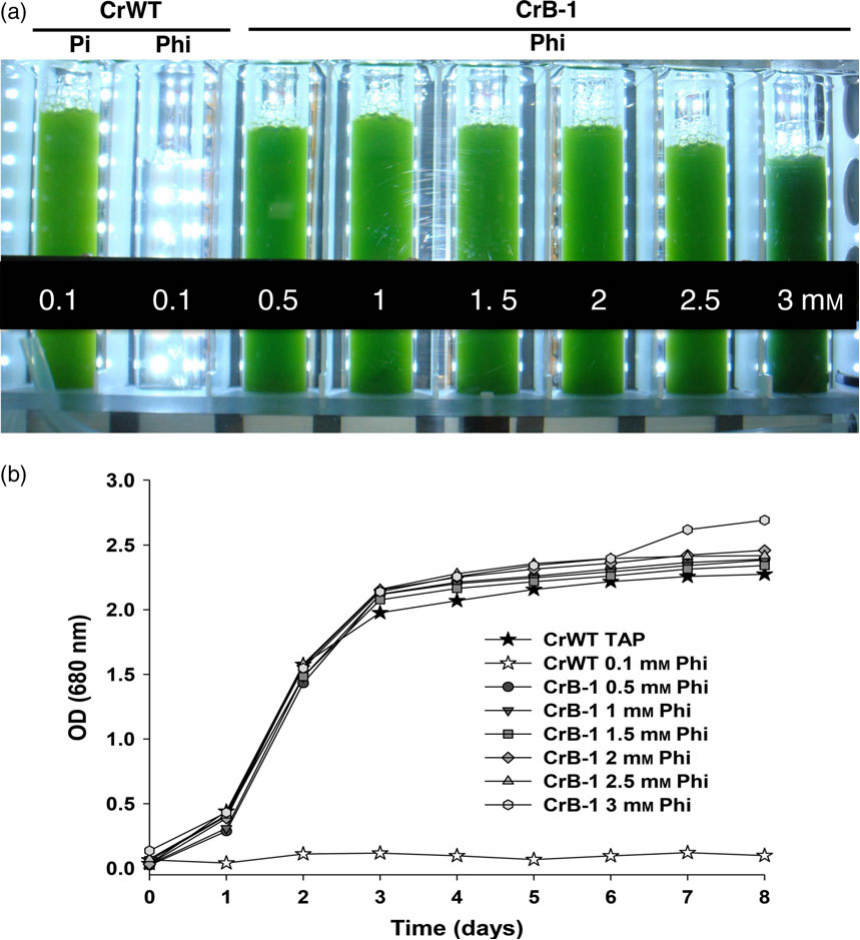
Growth of the CrB‐1 transgenic line in different concentrations of phosphite under sterile conditions. *Chlamydomonas reinhardtii* wild‐type CC‐125 (CrWT) was grown in Tris‐Acetate (TA) media supplemented with 0.1 mm phosphate (Pi) and phosphite (Phi), whereas the *C. reinhardtii* transgenic line (CrB‐1) was grown in TA media supplemented with 0.1, 0.5, 1, 1.5, 2, 2.5 and 3 mm Phi under sterile conditions. Phosphorus (P)‐replete and P‐starved cells were used as inoculum for the experiments with CrB‐1 and CrWT, respectively, in which the optical density (OD) at 680 nm was measured every day for 8 days. Cultures were performed using a photobioreactor (Multi‐Cultivator MC 1000) at a light intensity of 250 μmol photons/m^2^/s, 28 °C and bubbled with air. The CrWT strain was used as a control. The photograph shows the cultures 6 days after the inoculation.

It has been shown that high concentrations of Phi can inhibit plant and algae growth (Loera‐Quezada *et al*., 2015; Ticconi *et al*., 2001). To determine what was the optimum concentration for growth and also whether high concentrations were inhibitory, we performed time course experiments to measure cell growth using a range of Phi concentrations. For these experiments, we grew the CrB‐1 line in media containing 0.1, 0.5, 1, 1.5, 2, 2.5 and 3 mm Phi as the source of P, using the CrWT and 0.1 mm Pi as controls. As shown in Figure 3, the CrB‐1 line was able to grow in all Phi concentrations with similar growth kinetics as the WT strain using 0.1 mm Pi as a P source.

### *Chlamydomonas reinhardtii* lines capable of metabolizing Phi can outcompete other microalgae and naturally occurring contaminants

As mentioned above, managing contamination through metabolic engineering for the generation of microalgae capable of outcompeting undesirable microalgae and other microorganisms for restricted resources has been poorly studied. Therefore, to determine whether *C. reinhardtii* capable of metabolizing Phi has a competitive advantage to outgrow undesirable contaminants when Phi is used as the sole P source, we first designed experiments with the CrB‐1 transgenic line under nonsterile conditions using the Multicultivator photobioreactor. We found that the line CrB‐1 had limited growth in nonsterile media containing 0.1 mm Pi, probably as a consequence of bacterial contamination, which was observed since the first day after inoculation (Figure S1). In contrast, CrB‐1 grew vigorously in Phi containing media and overcame contamination under all treatments, particularly at 1 mm or higher Phi concentrations (Figure S1). Visual inspection of the different cultures (Figure S1a) correlated with cell growth as measured by OD (Figure S1b) and observations under a microscope (Figure S2). These experiments demonstrated that using microalgae capable of metabolizing Phi and Phi as the P source can be used to reduce the effect of biological contaminants naturally present in the water and reagents used to prepare media.

The next step was to determine whether microalgae able to metabolize Phi could outcompete other microalgae present in the growing system. Therefore, we performed competition experiments between the CrB‐1 transgenic strain and *Scenedesmus obliquus*. Previously, we reported that some microalgae (i.e.; *Botryococcus braunii* and *Ettlia oleoabundans*) were unable to metabolize Phi as a P source (Loera‐Quezada *et al*., 2015). As part of this work, we determined that other microalgae including *Haematococcus pluvialis* and *S. obliquus* were also unable to metabolize Phi (Figure S3). Taking advantage of the fact *S. obliquus* has a different morphology than *C. reinhardtii,* we selected *S. obliquus* as the microalgae competitor for these experiments.

**Figure 4:**
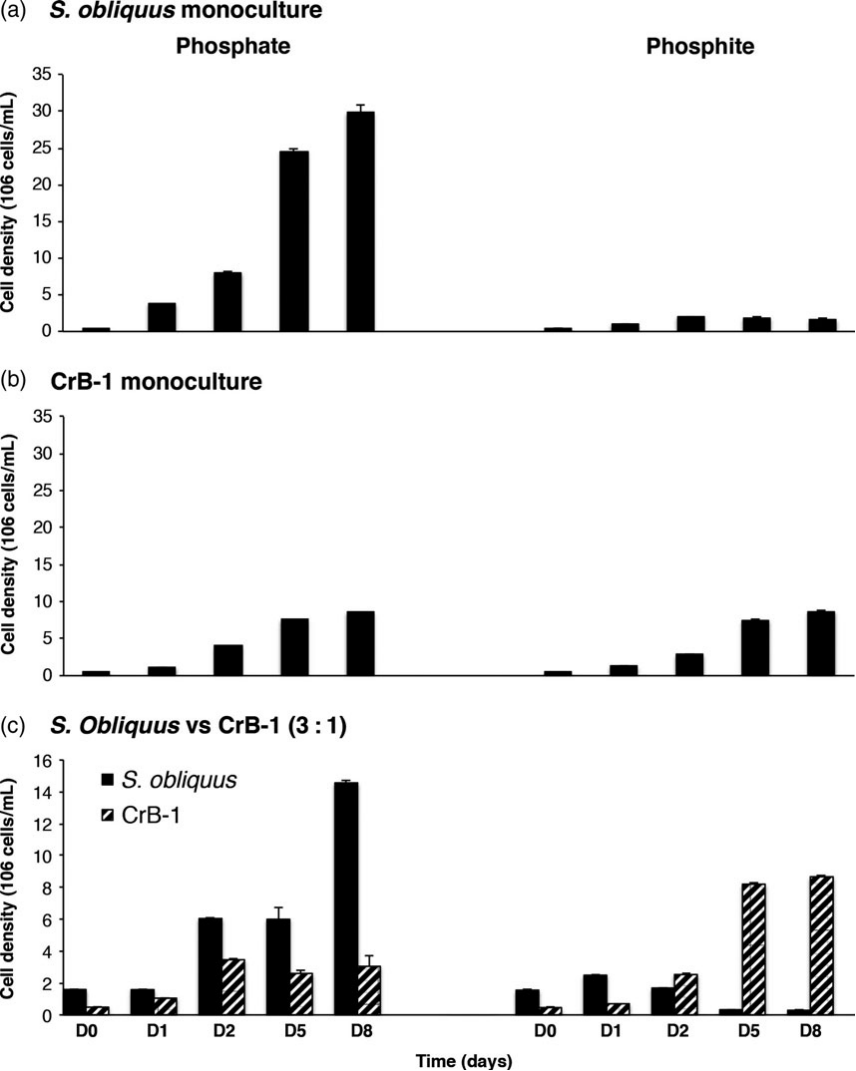
Growth competition experiments between the CrB‐1 transgenic line and *Scenedesmus obliquus* using medium supplemented with phosphite as the only phosphorus source. *Chlamydomonas reinhardtii* transgenic line (CrB‐1) and *S. obliquus* were grown in monocultures and mixed cultures using Tris‐Acetate (TA) medium supplemented with 0.1 mm phosphite (Phi) and 0.1 mm phosphate (Pi) as a phosphorus source. Inoculum of 3 : 1 of *S. obliquus*: CrB‐1 for the competition experiments was used. Cultures were performed using 50‐mL Erlenmeyer flasks at a light intensity of 50 μmol photons/m^2^/s and 28 °C.

In the competition experiments, we included monocultures of *C. reinhardtii* and *S. obliquus* as well as mixed cultures between these two microalgae to test the competitive capability of CrB‐1. We used inoculums of 1:1 and 3:1 of *S. obliquus*: CrB‐1 for the competition experiments. In the case of Pi treatments, we observed that *S. obliquus* grew to a much higher cell density than the CrB‐1 strain (Figure 4a). In media containing Phi as the sole source of P, CrB‐1 had a similar growth to that obtained in media supplemented with Pi, whereas the growth of *S. obliquus* in Phi containing media was severely reduced compared with that observed in media supplemented with Pi (Figure 4a,b). In the mixed cultures under Pi treatment, we observed that both strains had reduced growth compared with that achieved by monoculture growth and that *S. obliquus* is a better competitor as it achieved a cell density of approximately 5‐ to 6‐fold than that of the transgenic line CrB‐1 (Figure 4c). By contrast, in the case of Phi treatments, we observed that even when density of *S. obliquus* was greater than CrB‐1 at the initial inoculum, CrB‐1 proliferated faster than *S. obliquus* from the start of the experiment (Figure 4c), and after 5 days of growth of the experiments CrB1 completely outcompeted *S. obliquus,* achieving a cell density similar to that observed in the monoculture experiments (Figure 4c).

Similar results were obtained in a competition experiment between *H. pluvialis* and line CrB‐1 using a nonsterile system (Figure S4). In this competition experiments, we included monocultures of *C. reinhardtii* and *H. pluvialis*, as well as mixed cultures between these two microalgae to test the competitive capability of CrB‐1. We corroborated that *H. pluvialis* is unable to use Phi as P source and that the CrB‐1 strain metabolizes Phi efficiently, as we observed previously. CrB‐1 monocultures reached a normal growth with no external contamination interference (Figure S4), whereas in the mixed cultures, at 12 days after inoculation the competitive capability of the CrB‐1 over external contamination and *H. pluvialis* was evident (Figure S4).

**Figure 5:**
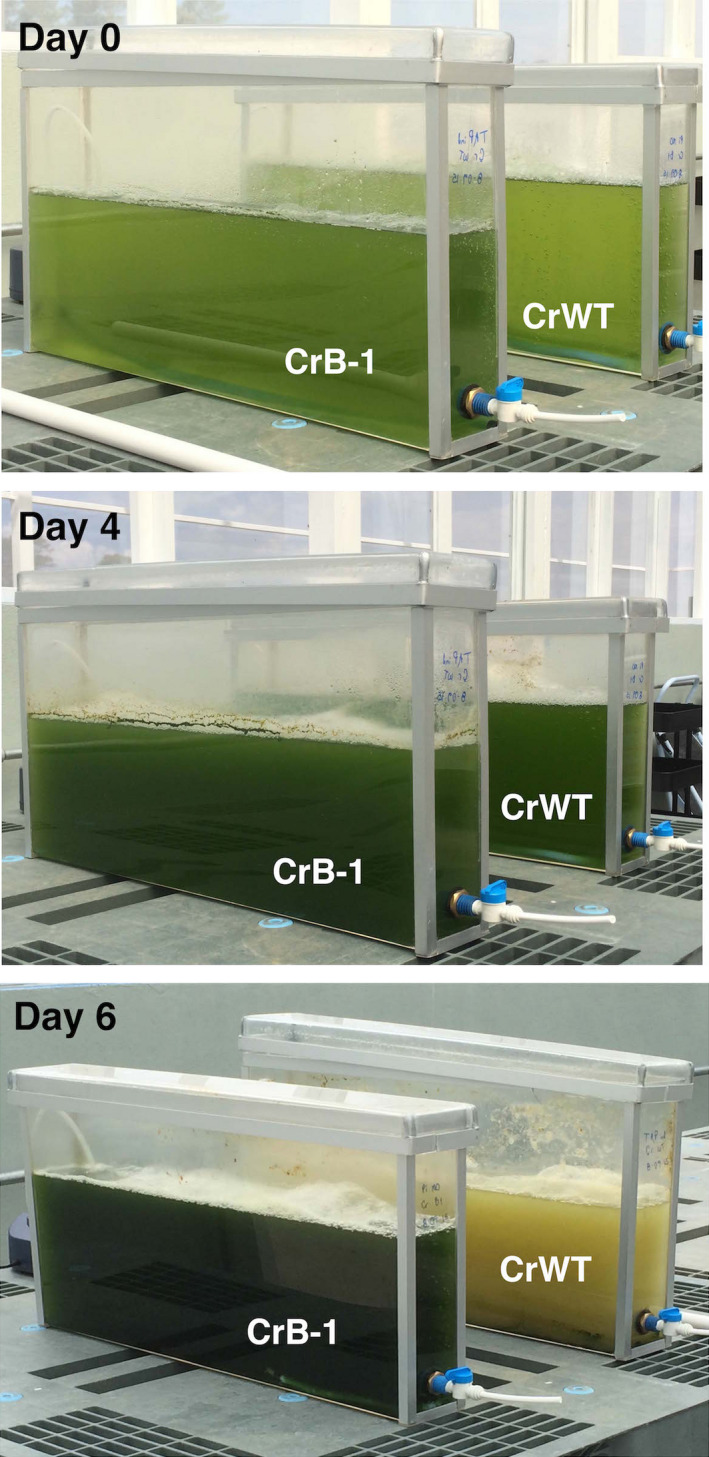
Growth of CrB‐1 in wall‐shaped bioreactors using phosphite as a phosphorus source under nonsterile conditions. *Chlamydomonas reinhardtii* transgenic line (CrB‐1) and the wild‐type CC‐125 (CrWT) were grown in Tris‐Acetate (TA) media prepared with industrial grade reagents, and supplemented with 0.1 mm phosphite (Phi) or phosphate (Pi) as a phosphorus (P) source. Cultures were performed using 10% (v/v) inoculum and wall‐shaped bioreactors under greenhouse conditions and bubbled with air.

Our data provided evidence that the system based on the *ptxD* gene and Phi as a P source has the potential to control external contamination, we performed experiments using wall‐shaped bioreactors to grow the CrB‐1 transgenic strain using Phi and the CrWT using Pi under nonsterile conditions. As shown in Figure 5, during the first 4 days, apparently both cultures looked similar. However, 6 days after inoculation, external contamination predominated over the CrWT strain and produced a white‐coloured culture, whereas the CrB‐1 line produced a green coloured, saturated culture, which continued to grow normally until day 16. Observations under the microscope allowed us to identify fungi and bacteria in the CrWT culture in Pi, which are likely the cause of the detriment to the culture.

## Discussion

Metabolic engineering of microalgae has been focused mainly on directly manipulating the pathway involved in the generation of the product of interest to increase the yield. Some of these efforts have attempted to improve lipid biosynthesis, carbon storage, bio‐hydrogen production and carotenoid production (Beer *et al*., 2009; Cordero *et al*., 2011; Liu *et al*., 2013; Trentacoste *et al*., 2013; Yu *et al*., 2011; Zeng *et al*., 2011). To date, biological and molecular strategies to control bacterial and microalgae contaminants that represent one of the major constraints for the production of microalgal biomass have received little attention (Kazamia *et al*., 2012; Shurin *et al*., 2013). Algal biotechnology companies that cultivate algae largely rely on the application of antibiotics and fungicides to control contamination (McBride *et al*., 2014). Community ecology approaches for cultivation in which the organism of interest grows in consortia with other carefully selected species have been advocated as an interesting alternative to improve both the productivity and stability of cultures (Kazamia *et al*., 2012, 2014). This has been supported by the observation that polycultures are more stable and productive tan monocultures under some scenarios of growth (Shurin *et al*., 2013). Furthermore, it has been shown that it is possible to use an environmental selective pressure to force consortia of microalgae to exhibit a certain train, such as increased lipid productivity (Mooij *et al*., 2013). These methods are attractive because they bypass chemical use and harness the dynamics of natural communities (Smith and Mcbride, 2015; Smith *et al*., 2010). However, it is not always possible or desirable to deal with algal consortia, especially when a particular algal strain or specific combination of microalgae species is required for a particular product. The approach presented here is an effective alternative, which bypassing the use of pesticides and fungicides, it also decreases the nutrient requirements for algal culture. Our results demonstrate that engineering microalgae to make them capable of metabolizing Phi is a feasible strategy to generate an effective system to control biological contaminants for the cultivation of microalgae, which may be complemented with biological and molecular strategies already developed. The engineered *C. reinhardtii* lines have the capacity to use Phi as a P source (Figures 1 and 3) and dominate the culture system in the presence of naturally occurring contaminants or even when deliberately inoculated with another algal species (Figures 4 and 5). Our findings are of relevance for closed photobioreactors because the major advantage of using Phi‐metabolizing strains is the possibility to avoid the need of using sterile conditions, which constitute one of the major costs in the operation of large photobioreactors**.** Moreover**,** one of the most important constraints for commercial production of microalgae using open culture systems is also the contamination caused by biological pollutants. This type of culture system is a more suitable to meet the requirements of large‐scale algae biomass production because of its low cost of maintenance and its relatively simple operation (Grobbelaar, 2012). However, open pond systems, such as raceways, are highly susceptible to bacteria, fungi, zooplankton or other undesirable microalgae (‘weeds’) contamination (Bínová *et al*., 1998; Chiaramonti *et al*., 2013; Gouveia, 2011; Lam and Lee, 2012; Letcher *et al*., 2013; Mahadevaswamy and Venkataraman, 1981; USDOE 2010; Verma *et al*., 2010). Therefore, the Phi‐based system has also a potential to solve contamination problems in open pond systems, extending its use to microalgae species different to those capable of growing in extreme environmental conditions.

Although the effectiveness of the system remains to be tested on a large‐scale and its utility for the generation of bioproducts still requires to be demonstrated, our results showed that, in principle, engineering the Phi metabolism is a biological advantage with a potential impact on current microalgae culture systems in the following ways: (i) it should allow a reduction in the operation costs of closed photobioreactors as the need for media and reactor sterilization would not be longer required; (ii) the system would make possible the extensive use of raceway ponds for the production of microalgae biomass and its derived products, which has been currently limited to a few microalgae species; (iii) the system could make possible the cultivation in open ponds of the numerous interesting microalgae species that currently are not being commercially exploited; (iv) the system should allow a reduction in the cost of production of biomass or industrially relevant compounds compared to the use of closed reactors as the system does not require the use of sterile conditions in the reactor or the media; (v) an improvement in yield and quality of the microalgae biomass and bioproduct is also expected, as contaminant organisms will be decreased or eliminated; and (vi) the Phi system should allow the metabolic engineering of different microalgae species to establish consortia to produce more complex mixes of compounds or even the use of microalgae and algae‐growth‐promoting bacteria, both capable of metabolizing Phi, to enhance biomass productivity and maintain specific combinations of desired species.

Because the system is based on the use of a new chemical compound as a P source for the transgenic microalgae, potential hazards need to be addressed with the use of this system to generate products for human and animal feeding. However, it is important to note that Phi is already widely used in agriculture as a fungicide and stimulant of plant growth and that the US Food and Drug Administration approved the use of Phi in many food and nonfood crops since 1997 because it is innocuous to humans and animals. Moreover, because Phi will be converted into Pi by PTXD inside the cells, there would be no concerns associated with the presence of a new compound inside the cells as it could be in the case of herbicides or antibiotics. Additionally, because Phi is also oxidized into Pi by the oxygen present in the atmosphere in a relatively short time (2–3 months), a negative environmental effect due to Phi discharge to industrial effluents is unlikely. These properties suggest that the Phi‐based system could be easily implemented to replace Pi fertilization in industrial cultures. Moreover, although there are no commercial formulations specifically designed for microalgae/cyanobacteria, sodium and calcium Phi salts are available in large quantities on the market at a lower price than fungicides, antibiotics and herbicides.

The availability of mineral nutrients is one of the most important limiting factors for both productivity and maintenance of microalgae cultures. The platform presented here could contribute to design fertilization strategies to optimize the use of nutrients, mainly of P, and avoid their excessive use, which may help to decrease eutrophication of water bodies if wastewater is discharged. This can be achieved taking advantage of the physicochemical properties of Phi, as for the case of Phi‐metabolizing plants (López‐Arredondo and Herrera‐Estrella, 2012). This is of special importance for algal biofuels production, because the projected requirements of P and nitrogen under the current scenario to produce biofuels would be untenable because of its drastic environmental impact (Shurin *et al*., 2013; Smith and McBride *et al*., 2014).

Transgenic microalgae and cyanobacteria have been considered by researchers and private companies as green cell‐factories with great potential to produce value‐added bioproducts. We suggest that the Phi‐based system represents a valuable tool to facilitate the development and competitiveness of biotechnological processes based on microalgae. As microalgae and cyanobacteria use Pi as the P source, this system should be easily implemented in any species amenable to methods of genetic modification. The challenge remains for the many species for which genetic transformation has not yet been developed. In these cases, we need to learn more about the organism of interest and to develop and optimize the molecular tools to introduce and express foreign genes. Alternatively, as the Phi‐metabolizing phenotype is very clear, this system could serve as a model to test also synthetic biology approaches.

## Experimental procedures

### Algal strain, growth medium and culture conditions

The green microalga *C. reinhardtii* wild‐type CC‐125 (mt +) was obtained from the Chlamydomonas Resource Center. This strain was maintained and cultured at 28 °C under continuous illumination of 50 μmol photons/m^2^/s in freshly prepared solid or liquid TAP medium (Tris Acetate Phosphate) (Gorman and Levine, 1965). Solid media were prepared using 1.5% (w/v) Bacto‐agar (Difco, Franklin Lakes, NJ). Liquid cultures were maintained in an orbital shaker (shaker incubator MRC LOM‐150DIG/500DIG), under the following culture conditions: temperature at 28 ± 2 °C, orbital shaking at 110 r min^−1^ and 50 ± 10 μmol photon/m^2^/s of continuous fluorescent white light.

*Scenedesmus obliquus C1S* (Do Nascimento *et al*., 2012) was a kind gift of Dr. Leonardo Curatti. This strain was maintained according to Do Nascimento *et al*. (2012) and cultured using similar conditions as for *C*. *reinhardtii* wild‐type CC‐125.

### Plasmid construction

The *ptxD* encoding gene from *P. stutzeri* WM88 (AF061070, http://www.ncbi.nlm.nih.gov/nuccore/AF061070) was codon‐optimized to the nuclear codon usage of *C. reinhardtii* according to the OptimumGene^™^‐Codon optimization Tool (GenScript, Piscataway, NJ) and placed under the control of the Hsp70A‐RbcS2 chimeric constitutive promoter of the pChlamy_4 vector (Life Technologies Corporation, Carlsbad, CA), which displayed the ble‐2A nuclear expression strategy previously reported (Rasala *et al*., 2012). The *ptxD* gene was synthesized with the required restriction sites and was then cloned into the *Eco*RI/*Xho*I and *Eco*RI/*Bgl*II sites to create the plasmids pChlamy_4*Xho*I and pChlamy_4*Bgl*II, respectively. The resulting plasmids were then subjected to restriction digestion analysis using *Nde*I/*Xho*I and sequencing analysis to corroborate the correct structure of the Hsp70A‐RbcS2::*ptxD* gene construct.

### *Chlamydomonas reinhardtii* nuclear transformations

The *C. reinhardtii* wild‐type (CrWT) strain used in this study was transformed by electroporation according to the user's guide of GeneArt^®^ Chlamydomonas TOPO^®^ Engineering Kits (Life Technologies Corporation). Briefly, cells of *C. reinhardtii* from the exponential phase were collected by centrifugation and resuspended in TAP medium supplemented with 40 mm sucrose. A total of 2 μg of *Sca*I‐linearized plasmid was added to the cells of each transformation, and 250 μL of the transformation mixtures was transferred into a 4‐mm electroporation cuvette and incubated at room temperature for 5 min. The electroporation parameters were set as follows: 600 V, 50 μF and resistance to infinity. After electroporation, cells were mixed with 5 mL of TAP‐40 mm sucrose solution and incubated for 24 h to let them recover from electroporation. Transformants were selected on TAP agar plates supplemented with 5 μg/mL zeocin as a selective agent. Screening of transformants for growth on Phi medium as the sole P source was carried out using multiwell culture plates and 50‐mL glass flasks. Selected transgenic lines were always maintained in Phi containing medium.

### PCR analysis

Genomic DNA was isolated as described by Newman and colleagues (Newman *et al*., 1990). The primers FWD*PTXD*Crein 5′‐CCTCGTCCGACTTCATCCTG‐3′ and REV*PTXD*Crein 5′‐GATGTGCGGCGT GAACAG‐3′ were used to amplify a *ptxD* fragment of 284 pb from 100 ng of genomic DNA as a template. The thermocycler was programmed as follows: 94 °C for 3 min, denaturation (94 °C for 30 s), annealing (64 °C for 30 s) and extension (72 °C 1 m) for 34 cycles, then a final extension at 72 °C for 7 min, with *Taq* DNA Polymerase Recombinant (Life Technologies). The PCR products were examined on a 1% agarose gel using SYBR‐Safe DNA gel stain (Life Technologies).

### Functional analysis of transgenic lines

To determine the ability of transgenic strains to grow using Phi as the sole P source, *C. reinhardtii* WT and the transgenic lines were grown in TA medium supplemented with potassium phosphite monobasic [KH_2_PO_3_, Wanjie International, CAS No. 13977‐65‐6] at different concentrations, ranging from 0.1 to 3 mm. Inocula from the different lines were in cultures previously grown in P‐free medium (1% v/v). These experiments were performed using a Multi‐Cultivator MC 1000 photobioreactor (Qubit Systems), which consisted of eight glass test tubes with a working volume of 80 mL each, bubbled with air and at a 28 °C temperature controlled by a thermostat water bath. Each tube was independently illuminated with white LEDs at 250 μmol photons/m^2^/s. Growth was estimated by measuring the optical density (680 nm).

### RT‐quantitative PCR analysis

Total RNA was isolated using the PureZol^™^ RNA isolation reagent (Bio‐Rad, Hercules, CA) following the manufacturer's instructions. The purified total RNA concentration was determined using a NanoDrop 2000 spectrophotometer (Thermo Scientific, Waltham, MA), and its quality and integrity were corroborated by agarose gel electrophoresis (1.5%). Subsequently, 10 μg of RNA was treated with DNase I New England Biolabs (Ipswich, MA) to eliminate any contaminating genomic DNA. A total of 4 μg of DNA‐free RNA was used for cDNA synthesis using the SuperScript III reverse transcriptase (Invitrogen; 10 min at 94 °C, 30 s at 94 °C, 30 s at 60 °C, and 40 s at 72 °C), following the manufacturer's instructions. Real‐time qPCR was performed in quadruplicates for each sample using a 7500 real‐time System and using the SYBR® Green Master Mix (Applied Biosystems, Carlsbad, CA) according to the manufacturer's instructions. The *C. reinhardtii* actin gene was used as a reference and was amplified in parallel with the *ptxD* gene using the specific primers (FWDACTCr 5′−cgaaatcgtgcgcgacatcaag−3′ and REVACTCr 5′−cgacagcacgatgttgttgtagaggtc−3′) and (TRCrFWD 5′−cacgacgagatcctgcagc−3′ and TRCrREV1 5′−aggaagtcagcgtccagccg−3), respectively. *Chlamydomonas reinhardtii* WT was used as an internal control. Standard curves were obtained using 4 five‐serial‐dilutions for *ptxD* and actin genes. Relative mRNA levels are expressed as the ratio of the genes of interest to the actin gene.

### Western blot analysis

For Western blot analysis, proteins were isolated from 50 mL of *C. reinhardtii* cells by centrifugation at 1700 ***g*** at 4 °C for 10 min in the late log phase. The pellet was washed with an osmotic buffer (MOPS 100 mm pH 7.3, sucrose 250 mm) and resuspended with 5 mL of the lysis buffer containing 50 PBS buffer pH 7.4, 5 mm β‐mercaptoethanol, 100 mm phenylmethylsulphonylfluoride (PMSF) and 1X protease inhibitor cocktail (Sigma Aldrich, St. Louis, MO). Cell suspension was sonicated with six short bursts of 30 s followed by intervals of 30 s for cooling on ice. After sonication, the supernatant was centrifuged at 23,897 ***g*** at 4 °C during 30 min. The resulting supernatants were quantified by Bradford reagent (Bio‐Rad) following the manufacturer's instructions. Samples representing 5 μg of total soluble protein were denatured at 95 °C for 3 min (in Laemmli buffer), separated in 12% of SDS‐PAGE gels and transferred to a PVDF membrane using a TransBlot (Thermo Scientific) at 25 V during 1 h. After blocking with 2.5% commercial milk, the membranes were probed with a monoclonal antibody diluted 1 : 250 (overnight, 4 °C). The membranes were then washed and incubated with a 1 : 1000 dilution of a horseradish peroxidase‐conjugated anti‐mouse IgG secondary antibody (Thermo Scientific) for 1–2 h. A working solution of ECL Western Blotting Substrate (Thermo Scientific) was prepared according to the manufacturer′s instructions and added to the membranes for 1 min. Membranes were analysed by chemiluminescent detection.

### PTXD activity determination

The enzymatic activity of PTXD was measured using *C. reinhardtii* cell extracts. A total of 50 mL of culture was pelleted by centrifugation at 4000 r.p.m. at 4 °C and was washed two times with 50% acetone to extract the chlorophyll. The resulting pellet was lysed by sonication as described above using a new resuspension buffer (50 mm of MOPS pH 7.3, 150 mm NaCl, 5% glycerol, 5 mm β‐mercaptoethanol, 100 mm phenylmethylsulphonylfluoride (PMSF) and 1X protease inhibitor cocktail (Sigma Aldrich). Supernatants were quantified with Bradford reagent, extracts were adjusted to the same concentration of protein (0.5 μg/μL) and then PTXD enzymatic activity was evaluated using a modified protocol described by Berkowitz *et al*. (2011). Modifications included resazurin at a 5 mm final concentration and an incubation time of 16 h.

### Competition assay

*Scenedesmus obliquus C1S* (Do Nascimento *et al*., 2012) was used as one of the competitive microalgae. For experiments, P‐depleted cells at 1 : 1 and 3 : 1 (*S. obliquus*:* C. reinhardtii* transgenic lines CrB‐1) inoculum proportions were cultivated in TA media supplemented with 0.1 mm Phi and 0.1 mm Pi. Monocultures of both microalgae were used as controls.

Because *H. pluvialis* (UTEX 2505) predominantly uses nitrate (NO_3_^−^) as the nitrogen source (Göksan *et al*. 2011), whereas *C. reinhardtii* uses ammonium (NH_4_^+^) (Camargo *et al*. 2007), in the case of competition experiments using both microalgae, the TAP medium was modified (TAPMod) accordingly to meet the requirements of both at the same time. Therefore, ammonium chloride (NH_4_Cl) was substituted by ammonium nitrate (NH_4_NO_3_) at the same molarity (280.4 mm). As we observed that the growth of *H. pluvialis* in TAPMod was slightly decreased in comparison to *C. reinhardtii* transgenic strains, to avoid an advantage of *C. reinhardtii* strains over *H. pluvialis*, we decided to use a higher inoculum of the competitive microalgae. Competition experiments were performed using 0.01% and 0.001% (v/v) as the inoculum of the transgenic strain (CrB1), whereas 3% (v/v) was used as inoculum of *H. pluvialis*. Monocultures of each species were used as controls, which were grown in parallel with mixed cultures. Therefore, monocultures and mixed cultures of the transgenic strain and *H. pluvialis* were established as follows: CrB10.01 (inoculum 0.01%), CrB10.001 (inoculum 0.001%), *H. pluvialis*‐3 (inoculum 3%), CrB10.01 +  *H. pluvialis*‐3 (inoculum 0.01 + 3%) and CrB10.001 +  *H. pluvialis*‐3 (inoculum 0.001 + 3%), using the TAPMod medium supplemented with KH_2_PO_3_ and KH_2_PO_4_ to a final concentration of 0.1 mm.

Experiments were carried out using 50‐mL glass flasks under the culture conditions described above. Each treatment was performed in duplicate. The algal growth was estimated by OD measuring (750 nm) and by cell counting (Neubauer chamber). For experiments under nonsterile conditions, sterilization of materials and growth media were avoided to allow microorganisms to invade the axenic cultures.

## Supporting information


**Figure S1.** Growth of the CrB‐1 transgenic line in different phosphite concentrations under non‐sterile conditions. *Chlamydomonas reinhardtii* transgenic line (CrB‐1) was grown in Tris‐Acetate (TA) media supplemented with 0.1, 1, 2, 3, 4, and 5 mm phosphite (Phi) under non‐sterile conditions. The controls used were 0.1 mm phosphate (Pi) and non‐inoculated treatments (Ni). Cultures were performed using a photobioreactor (Multi‐Cultivator MC 1000) at a light intensity of 250 µmol photons/m^2^/s, 28 °C and bubbled with air, and the optical density (OD) at 680 nm was measured every day for 9 days. The photograph shows the cultures 6 days after inoculation.

**Figure S2.** Control of contaminants in media supplemented with phosphite as a phosphorus source. *Chlamydomonas reinhardtii* transgenic line (CrB‐1) was grown in Tris‐Acetate (TA) media supplemented with different phosphite (Phi) concentrations (0.1, 1, 2, 3, 4, and 5 mm) and 0.1 mm phosphate (Pi) under non‐sterile conditions, using non‐inoculated treatment as a control. The photographs show the cultures grown under: (a) 0.1 mm Pi, (b) 0.1 mm Phi and (c) 5 mm Phi, 6 days after inoculation. *Chlamydomonas reinhardtii* cells have green coloration, whereas contaminant organisms (arrowed) have no colour. Cultures were performed using a photobioreactor (Multi‐Cultivator MC 1000) at a light intensity of 250 µmol photons/m^2^/s, 28 °C and bubbled with air.

**Figure S3.** Growth of *Haematococcus pluvialis* and *Scenedesmus obliquus* in media supplemented with phosphite as a phosphorus source. *Scenedesmus obliquus* and *Haematococcus pluvialis* were cultured in Tris‐Acetate (TA) media supplemented with 0.1 mm phosphite (Phi) as a phosphorus source for 16 days. Cultures were performed using 10% (v/v) using 50 mL Erlenmeyer flasks at a light intensity of 50 µmol photon/m^2^/s and 28 °C. *Chlamydomonas reinhardtii* transgenic line (CrB‐1) was used as a control.

**Figure S4.** Growth competition experiments between the CrB‐1 transgenic line and *Haematococcus pluvialis* using medium supplemented with phosphite as the only phosphorus source under non‐sterile conditions. *Chlamydomonas reinhardtii* transgenic line (CrB‐1) and *Haematococcus pluvialis* were grown in monocultures and mixed cultures using TA medium supplemented with 0.1 mm phosphite (Phi) and 0.1 mm phosphate (Pi) as a phosphorus source. CrB‐1 cultures were started with 0.01% or 0.0001% inoculum, whereas *H. pluvialis* cultures were always used at 3% (v/v) either for monocultures or mixed cultures. Cultures were performed using a 50 mL Erlenmeyer flask at a light intensity of 50 µmol photons/m^2^/s and a temperature of 28 °C. In the figure on top of each flask the strain inoculated and the type of inoculum are indicated. The top panel shows the single or mixed cultures grown in media containing phosphate as a sole P source and in the bottom panel the culture in media containing phosphite as a sole P source. In all cases the media and flasks were not sterilized.
