# Supplementary figures and images for: A novel genetic engineering platform for the effective management of biological contaminants for the production of microalgae
Source: Plant Biotechnol J. 2016 May 28;14(10):2066–76. doi: 10.1111/pbi.12564 (PMC5043480; doi:10.1111/pbi.12564)

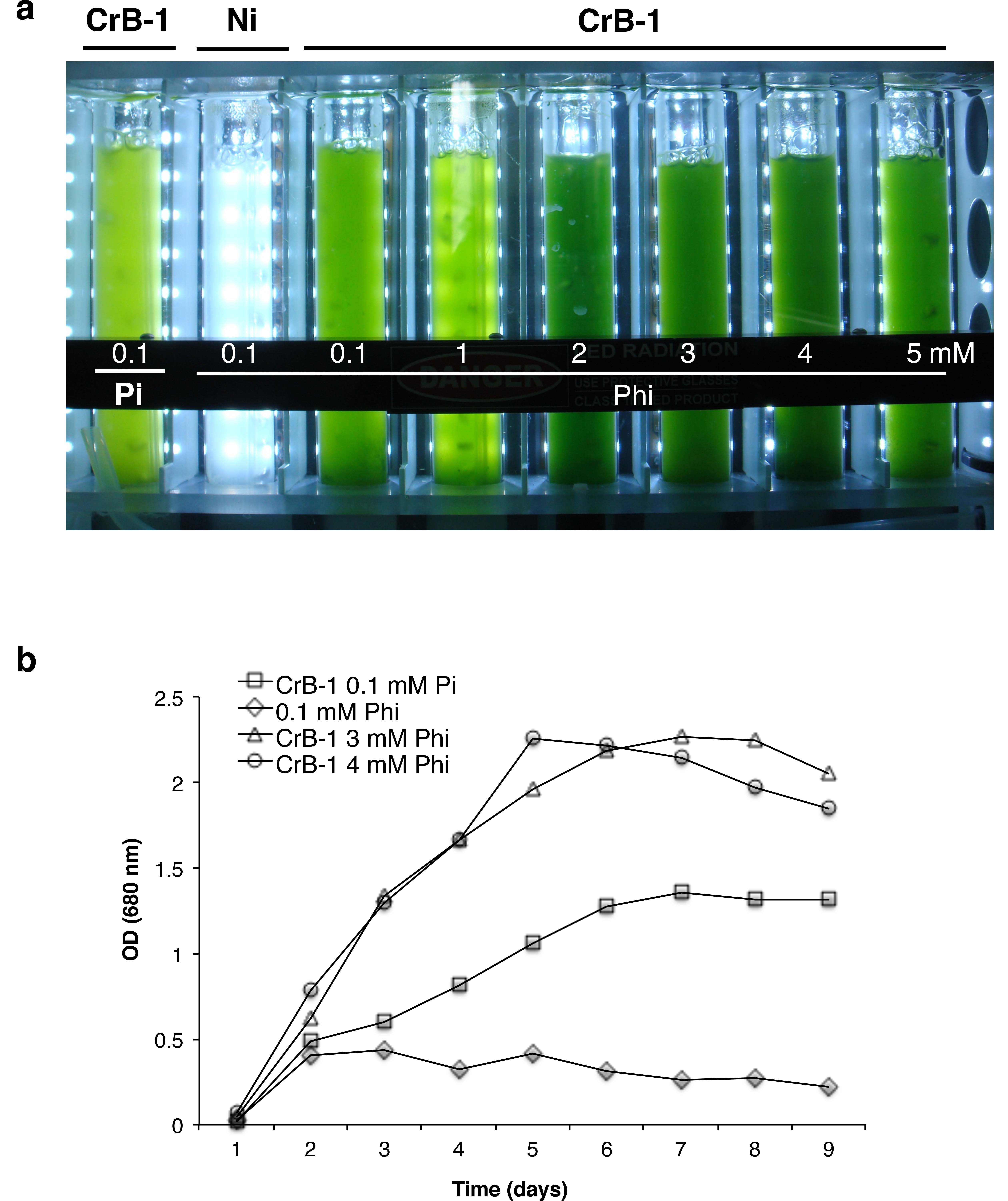

Supplement: Supplementary file 1 — Figure S1. Growth of the CrB‐1 transgenic line in different phosphite concentrations under non‐sterile conditions. Chlamydomonas reinhardtii transgenic line (CrB‐1) was grown in Tris‐Acetate (TA) media supplemented with 0.1, 1, 2, 3, 4, and 5 mm phosphite (Phi) under non‐sterile conditions. The controls used were 0.1 mm phosphate (Pi) and non‐inoculated treatments (Ni). Cultures were performed using a photobioreactor (Multi‐Cultivator MC 1000) at a light intensity of 250 µmol photons/m2/s, 28 °C and bubbled with air, and the optical density (OD) at 680 nm was measured every day for 9 days. The photograph shows the cultures 6 days after inoculation. [file PBI-14-2066-s003.jpg]

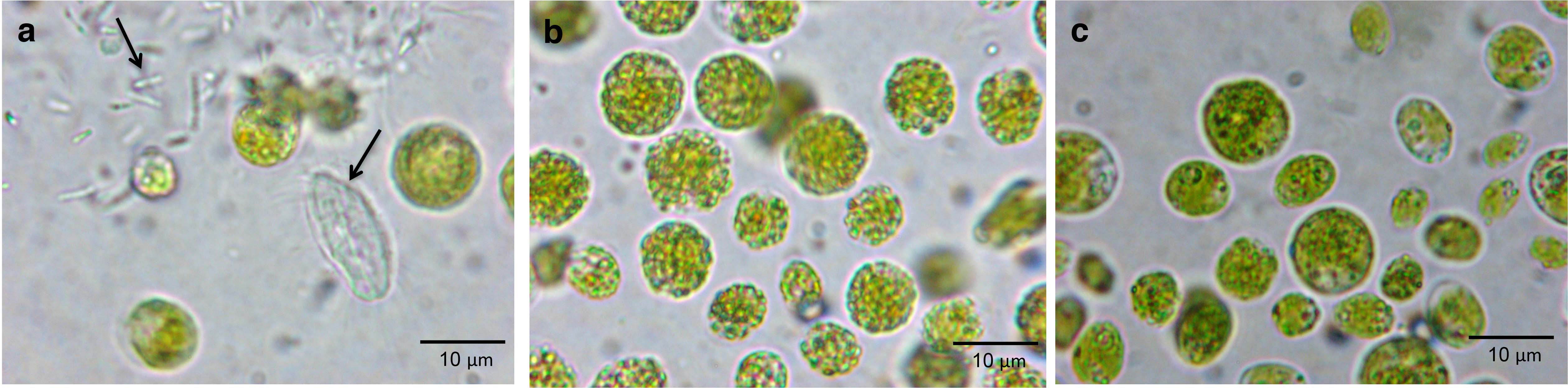

Supplement: Supplementary file 2 — Figure S2. Control of contaminants in media supplemented with phosphite as a phosphorus source. Chlamydomonas reinhardtii transgenic line (CrB‐1) was grown in Tris‐Acetate (TA) media supplemented with different phosphite (Phi) concentrations (0.1, 1, 2, 3, 4, and 5 mm) and 0.1 mm phosphate (Pi) under non‐sterile conditions, using non‐inoculated treatment as a control. The photographs show the cultures grown under: (a) 0.1 mm Pi, (b) 0.1 mm Phi and (c) 5 mm Phi, 6 days after inoculation. Chlamydomonas reinhardtii cells have green coloration, whereas contaminant organisms (arrowed) have no colour. Cultures were performed using a photobioreactor (Multi‐Cultivator MC 1000) at a light intensity of 250 µmol photons/m2/s, 28 °C and bubbled with air. [file PBI-14-2066-s002.jpg]

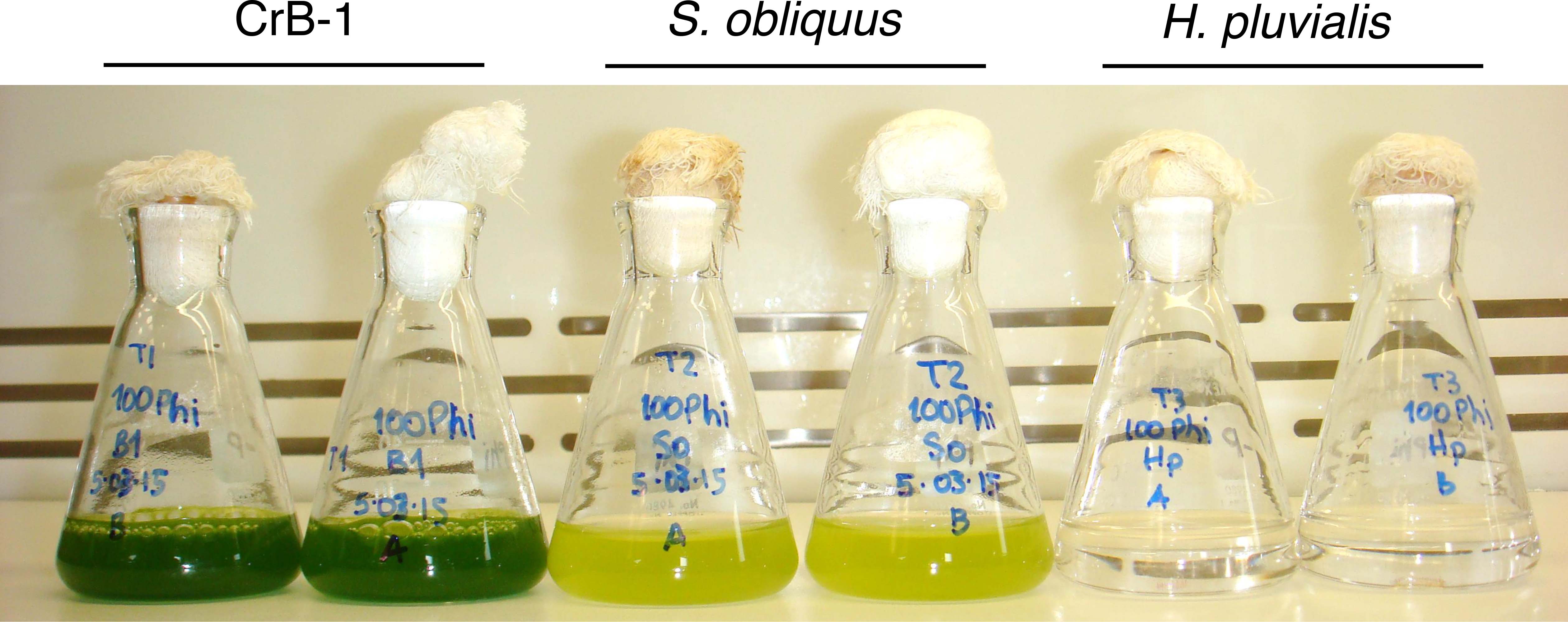

Supplement: Supplementary file 3 — Figure S3. Growth of Haematococcus pluvialis and Scenedesmus obliquus in media supplemented with phosphite as a phosphorus source. Scenedesmus obliquus and Haematococcus pluvialis were cultured in Tris‐Acetate (TA) media supplemented with 0.1 mm phosphite (Phi) as a phosphorus source for 16 days. Cultures were performed using 10% (v/v) using 50 mL Erlenmeyer flasks at a light intensity of 50 µmol photon/m2/s and 28 °C. Chlamydomonas reinhardtii transgenic line (CrB‐1) was used as a control. [file PBI-14-2066-s004.jpg]

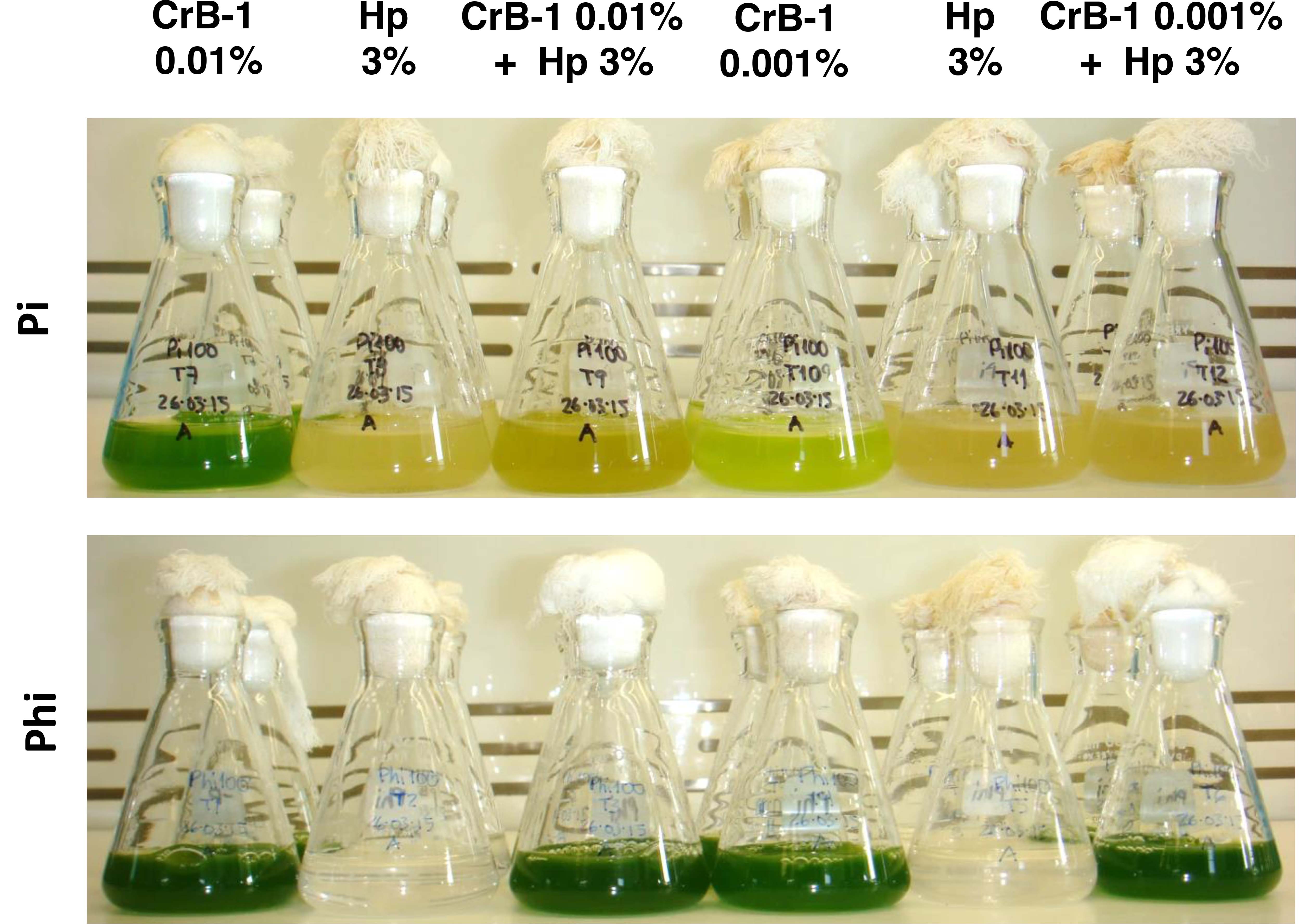

Supplement: Supplementary file 4 — Figure S4. Growth competition experiments between the CrB‐1 transgenic line and Haematococcus pluvialis using medium supplemented with phosphite as the only phosphorus source under non‐sterile conditions. Chlamydomonas reinhardtii transgenic line (CrB‐1) and Haematococcus pluvialis were grown in monocultures and mixed cultures using TA medium supplemented with 0.1 mm phosphite (Phi) and 0.1 mm phosphate (Pi) as a phosphorus source. CrB‐1 cultures were started with 0.01% or 0.0001% inoculum, whereas H. pluvialis cultures were always used at 3% (v/v) either for monocultures or mixed cultures. Cultures were performed using a 50 mL Erlenmeyer flask at a light intensity of 50 µmol photons/m2/s and a temperature of 28 °C. In the figure on top of each flask the strain inoculated and the type of inoculum are indicated. The top panel shows the single or mixed cultures grown in media containing phosphate as a sole P source and in the bottom panel the culture in media containing phosphite as a sole P source. In all cases the media and flasks were not sterilized. [file PBI-14-2066-s001.jpg]
